# Tumor Cell Resistance to the Inhibition of BRAF and MEK1/2

**DOI:** 10.3390/ijms241914837

**Published:** 2023-10-02

**Authors:** Wenjing Chen, Jong-In Park

**Affiliations:** Department of Biochemistry, Medical College of Wisconsin, Milwaukee, WI 53226, USA; wchen@mcw.edu

**Keywords:** BRAF, MEK, tumor, drug resistance

## Abstract

*BRAF* is one of the most frequently mutated oncogenes, with an overall frequency of about 50%. Targeting BRAF and its effector mitogen-activated protein kinase kinase 1/2 (MEK1/2) is now a key therapeutic strategy for *BRAF*-mutant tumors, and therapies based on dual BRAF/MEK inhibition showed significant efficacy in a broad spectrum of *BRAF* tumors. Nonetheless, BRAF/MEK inhibition therapy is not always effective for *BRAF* tumor suppression, and significant challenges remain to improve its clinical outcomes. First, certain *BRAF* tumors have an intrinsic ability to rapidly adapt to the presence of BRAF and MEK1/2 inhibitors by bypassing drug effects via rewired signaling, metabolic, and regulatory networks. Second, almost all tumors initially responsive to BRAF and MEK1/2 inhibitors eventually acquire therapy resistance via an additional genetic or epigenetic alteration(s). Overcoming these challenges requires identifying the molecular mechanism underlying tumor cell resistance to BRAF and MEK inhibitors and analyzing their specificity in different *BRAF* tumors. This review aims to update this information.

## 1. Introduction

*BRAF* is one of the most frequently mutated oncogenes, with an overall frequency of about 50%. Commonly occurring *BRAF* mutations switch the codon usage in the activation segment of the kinase domain, such as Val600 to Glu, Lys, or Asp, and render the kinase constitutively active independently of its upstream activator RAS, thereby causing the hyperactivation of its downstream effector, the MEK-extracellular signal-regulated kinase (ERK) cascade. Among the *BRAF* mutations identified thus far, *BRAF*^V600E^ is most common with frequencies ~50% in melanomas, ~40% of papillary thyroid carcinomas, ~10% of colorectal cancers, ~5% of lung adenocarcinomas while also being detected in a subset of brain and hematological malignancies (Table 1). As such, there has been much effort to develop small molecule inhibitors that selectively target BRAF and its effector cascade MEK/ERK, and many inhibitors have been successfully developed [1,2]. Indeed, BRAF inhibitor (BRAFi) treatment resulted in high response rates in patients. However, the rates were short-lived due to the development of therapy resistance, which involves mainly the reactivation of the MEK/ERK cascade [3,4,5,6]. Subsequently, a MEK inhibitor (MEKi) was combined with BRAFi, significantly extending the median duration of response [4,5]. Since then, increasing evidence supports that dual BRAF/MEK inhibition improves clinical outcomes compared with BRAF inhibition alone in different *BRAF*^V600E^ tumors [7].

**Figure 1:**
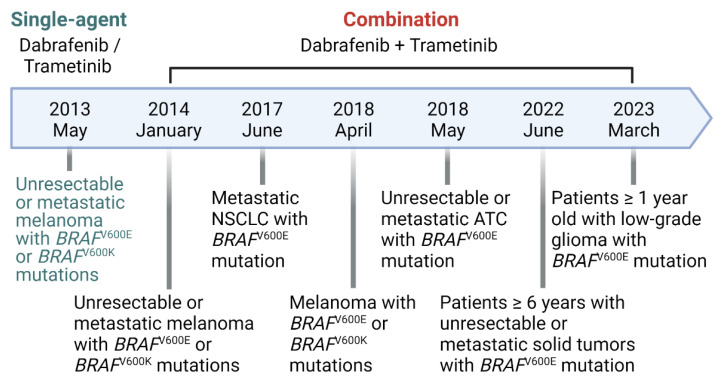
The history of FDA approval of dabrafenib and trametinib. Dabrafenib and trametinib have been approved by the FDA for monotherapy and combination therapy for *BRAF*^V600^ mutant solid tumors.

Combining the BRAFi dabrafenib (TAFINLAR^®^) and the MEKi trametinib (MEKINIST^®^) is an effective dual BRAF/MEK inhibition for cancer therapy, and the U.S. Food and Drug Administration (FDA) has approved these drugs for mono- and combination-therapy (Figure 1). A dabrafenib and trametinib combination (hereafter D/T combination) significantly improved response rates (76% vs. 54%), prolonged progression-free survival (PFS; 9.4 versus 5.8 months), and reduced skin toxicities compared with dabrafenib monotherapy in BRAF melanoma patients [4]. FDA recently granted approvals to the D/T combination therapy for patients with melanoma with *BRAF*^V600E^ or *BRAF*^V600K^ mutations [23] and those with metastatic anaplastic thyroid cancer [24] and non-small cell lung cancer (NSCLC) with *BRAF*^V600E^ mutation [25]. The D/T combination therapy also modestly improved response rates compared to BRAFi monotherapy (12% vs. 5%) in *BRAF*^V600E^ colon cancer patients [26,27]. However, it is not indicated for patients with colorectal cancer because of the relatively high intrinsic resistance of the tumor type. More recently, the FDA approved D/T combination for the treatment of adult and pediatric patients (≥ six years of age) with unresectable or metastatic solid *BRAF*^V600E^ tumors who have progressed following prior treatment and have no satisfactory alternative treatment options [28] and for pediatric patients (≥ one year of age) with low-grade *BRAF*^V600E^ glioma who require systemic therapy [29]. Nevertheless, the D/T combination is not always effective for *BRAF* tumors because certain *BRAF* tumors have an intrinsic ability to rapidly adapt to the presence of the drugs by bypassing drug effects via rewired signaling/metabolic/regulatory networks. Moreover, almost all tumors initially responsive to D/T combination therapy eventually acquired therapy resistance via an additional genetic/epigenetic alteration(s). Understanding the similarities and differences in therapy resistance in different tumor types is crucial. The goal of this review is to update this information.

## 2. MEK/ERK-Dependent Resistance Mechanisms 

**Figure 2:**
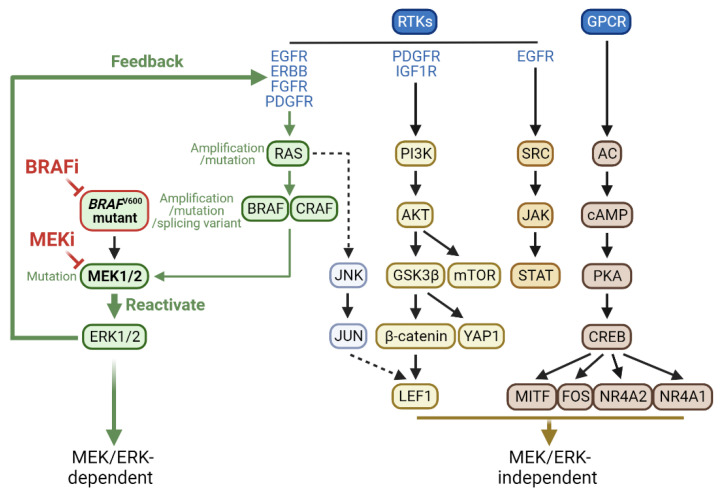
Intracellular mechanisms of tumor cell resistance to BRAFi and MEKi. Tumor cells can develop resistance to BRAFi and MEKi mainly by reactivating the MEK/ERK pathway by altering the regulators and molecular switches in the RAS/RAF/MEK pathway. Tumor cells can also develop drug resistance in an MEK/ERK-independent manner through various pathways illustrated. This figure was created with biorender.com.

Many therapy resistance mechanisms have been reported from BRAFi monotherapy cases [30,31,32,33]. ERK1/2 reactivation has been identified as a primary mechanism of BRAFi resistance in BRAF^V600E^ melanoma, colon, and thyroid cancers [34,35]. Accordingly, combining BRAFi with an inhibitor of MEK1/2 or ERK1/2 was evaluated to prevent the MEK/ERK reactivation. Indeed, concomitant inhibition of ERK1/2 or MEK1/2 has been shown to attenuate BRAFi resistance in different cell lines and preclinical models, providing a rationale for dual BRAF/MEK inhibition for therapy [36,37,38]. Although this strategy is successful, ERK1/2 reactivation remains a significant resistance mechanism in the combination therapy. In addition, MEK/ERK-independent resistance mechanisms are also activated, albeit at a lower frequency. These mechanisms are described below, illustrated in Figure 2, and summarized in Table 2 and Table 3.

### 2.1. MEK/ERK-Dependent Adaptive Resistance 

Specific tumor cells can rapidly adapt to the presence of BRAF/MEK inhibitors by turning on a feedback mechanism that can reestablish MEK/ERK signaling, which is often mediated through a receptor tyrosine kinase (RTK) signaling pathway (illustrated in Figure 2). The rates of adaptive resistance development are varied in cancers, and relatively low in melanoma compared to colon and thyroid cancers. For example, the relatively low efficacy of vemurafenib/PLX4032 in *BRAF*^V600E^ colon cancer is mainly attributed to the ability of the tumor cells to rapidly feedback-upregulate epidermal growth factor receptor (EGFR) signaling in response to the BRAFi, which does not occur as effectively in melanoma cells due to their intrinsically low EGFR expression [69]. Similarly, *BRAF*^V600E^ thyroid cancer cells can rapidly relieve the negative feedback-regulation of *human epidermal growth factor receptor 3* (*HER3/ErbB3*) transcription and increase autocrine secretion of the HER2 and HER3 ligand neuregulin 1 in response to vemurafenib, which also does not occur as effectively in melanoma cells [68]. This is accompanied by the rebound of ERK1/2 activity, which the HER kinase inhibitor lapatinib prevented [68]. Lapatinib also sensitized these tumor cells to vemurafenib [68]. Although HER3 is also activated through transcriptionally increased neuregulin in *BRAF*^V600E^ melanoma cell lines following exposure to BRAFi and/or MEKi, HER3 activation mainly leads to protein kinase B (AKT) hyperphosphorylation. Antibodies directed against different HER3 surface epitopes prevented the establishment of resistance to BRAF/MEK inhibitors [74]. Melanoma cells mainly acquired adaptive resistance to vemurafenib via platelet-derived growth factor receptors-β (PDGFR-β) upregulation [53]. Therefore, cellular context-dependent heterogeneity can determine the efficacy of a therapy.

### 2.2. MEK/ERK-Dependent Acquired Resistance 

Alterations of the molecular switches in the Ras/Raf/MEK/ERK pathway have been the primary mechanism of acquired resistance involving pathway reactivation (Figure 2). Various alterations of these switches have been detected in a tumor-specific manner, mainly in skin, colon, and thyroid cancers, as summarized below. While these alterations may develop because of the selection pressure of inhibitors, some of them may preexist in tumor cells and become dominant upon the selection pressure.

Alterations at RAS and upstream regulator level: BRAFi-resistance of *BRAF^V600E^* tumor cells is mainly associated with ERK1/2 reactivation. Intriguingly, earlier versions of BRAFi can drive BRAF^V600E^ binding to wild-type BRAF or CRAF, RAS-dependent wild-type RAF activation, and subsequently MEK/ERK activation [75,76,77]. The emergence of *RAS* mutation or amplification often facilitated MEK/ERK reactivation via these mechanisms. For example, *NRAS* mutations such as *NRAS*^Q61K^ and *NRAS*^A146T^ were detected in dabrafenib-resistant melanoma patient tumors and cell lines [37,53]. Similarly, activating mutations on different *RAS* isoforms, such as *KRAS*^G12V^, *NRAS*^Q61K^, and *NRAS*^G13D^, were also detected in dabrafenib-resistant thyroid cancers of patients [78]. In addition, increased *NRAS* expression was also found in *BRAF*^V600E^ vemurafenib-resistant melanoma cell lines [65]. Of note, *KRAS* amplification and emergence of *KRAS*^G12C^ in cell free DNA have been detected in D/T combination-resistant colon cancer patients, albeit at a much lower frequency than in BRAFi monotherapy [38,44], which suggests that trametinib cannot completely suppress the emergence of MEK/ERK-dependent therapy resistance in cancers. 

Alterations at BRAF level: *BRAF* splicing variation and amplification have been detected in D/T combination therapy-resistant melanoma in patients [39,40]. *BRAF* amplification has also been associated with D/T combination resistance in colon cancer patients [38,44]. A novel *BRAF* splicing isoform lacking exons 2-10 was detected in one out of five patients with D/T combination resistant melanoma tumors and that was undetectable in the pre-treatment tumor [40]. Similarly, in-frame deletion mutations involving exons 2-8, which includes the Ras-binding domain, were also detected in D/T combination-resistant *BRAF* melanomas in patients, albeit at a low frequency of 0.4% [41]. BRAF-activating deletion mutations were also detected at low frequencies (0.6–1%) in pancreatic, lung, ovarian, and thyroid tumors [79,80]. These deletions shorten the β3/αC-helix loop of BRAF and hinder its flexibility by locking the helix in the active αC-helix-in conformation that favors dimer formation [79,80]. The influence of the β3-αC deletion mutation on the binding profiles of three BRAF inhibitors (AZ628, dabrafenib, and vemurafenib) indicated that the β3-αC deletion mutation enhances the flexibility of the αC helix and alters structural conformation, which weakens the interactions between BRAF and these inhibitors [81]. 

Alterations at MEK1/2 level: *MEK2* mutations such as *MEK2*^C125S^ and *MEK2*^Q60P^ have been detected at higher frequencies in D/T combination therapy-resistant melanoma of patients than in BRAFi- or MEKi-monotherapy-resistant tumors [39,40]. Interestingly, *MEK1* mutations were detected at a lower frequency in these studies, and only *MEK2*^C125S^, but not the synonymous *MEK1*^C121S^, conferred resistance to the D/T combination [39,40]. Nonetheless, *MEK1*^C121S^ exhibited increased kinase activity and conferred resistance to RAF and MEK inhibitors in melanoma cell cultures [82]. Of note, an *in vitro* screening revealed that a mutation on the allosteric drug binding pocket or αC-helix of MEK confers resistance to allosteric MEK inhibition, and, consistent with this, MEKi-resistant *MEK1*^P124L^ mutation was detected in selumetinib/AZD6244-resistant *BRAF*^V600E^ melanoma of patients [71]. In colon cancer, *MEK1*^F53L^ mutation was detected in D/T combination-resistant tumor biopsies, albeit at lower frequencies [38]. However, *MEK1* or *MEK2* alterations were not detected in human colon cancer cell lines that have developed MEKi resistance in vitro [70]. These observations suggest that trametinib is the main selection pressure driving *MEK1* and *MEK2* mutations in tumor cells treated with D/T-combination and that these kinases may have a functional difference in therapy-resistance.

Alterations at ERK1/2 level: MEK1/2 are considered the only ERK1/2 activators, and, in that context, a constitutively active *ERK* mutation would be an effective strategy for tumor cells to bypass the effects of MEK1/2 inhibition. Nevertheless, D/T combination therapy-resistant *ERK1/2* mutations have rarely been reported. Of note, unlike MEK1/2 or most other kinases, the threonine–glutamic acid–tyrosine residue (*TEY*) site in the activation loop of ERK1/2 cannot be replaced by phosphomimetic amino acids to generate a constitutively active mutant [83]. Autophosphorylation is the only way for ERK to increase its activity autonomously, and its rate can increase upon several synergistic mutations that facilitate hydrogen bonding between the phosphoryl acceptor and catalytic nucleophile and different mutations that affect the gatekeeper residue [84,85,86]. Nevertheless, these ERK mutants display substantially lower activity than MEK1/2-activated ERK and produce limited effects in cells that are not cell-proliferative [87,88]. Considering this, constitutively active *ERK* mutation, but not other *ERK* mutations that affect ERK interaction with MEK1/2, phosphatases, or scaffolds, is probably not a feasible strategy for tumor cells to resist BRAF and MEK1/2 inhibition. Of note, different *ERK* mutations arise in ERK inhibitor-resistant tumor cells in culture, and it is important to understand by what mechanism these mutations facilitate restoring ERK activity in the tumor cells [89].

Interestingly, the combination of trametinib with the BRAFi PLX4720 induced ERK1/2 translocation to endoplasmic reticulum in BRAF mutant melanoma cells, and the protein kinase R-like endoplasmic reticulum kinase (PERK) phosphorylated ERK1/2 upon exiting endoplasmic reticulum [47]. Activated ERK1/2 via this mechanism phosphorylated activating transcription factor 4 to activate cytoprotective autophagy, eventually driving resistance to dual BRAF and MEK1/2 inhibition [47]. A separate study also reported the involvement of PERK-mediated ERK1/2 activation in BRAFi resistance [67]. Consistent with this, upregulation of glucose-regulated protein 78 and phosphorylation of activating transcription factor 4 were detected in tumors of patients resistant to PLX4720 and trametinib combination [47]. This suggests that certain tumor cells can activate ERK1/2 via a non-canonical mechanism. It is important to address whether similar non-canonical mechanisms may exist and decrease the efficacy of the D/T combination and whether these mechanisms may vary in tumors and underlie tumor-specific heterogeneous outcomes of the therapy. Since dual BRAF and ERK1/2 inhibition effectively abrogates clonal outgrowth of *BRAF*^V600E^ colorectal cancer cells, which have relatively high intrinsic resistance to BRAFi/MEKi combination [49], the addition of ERK inhibition to the combination strategy is promising. As such, predicting possible bypass mechanisms at ERK1/2 level is critical. Advanced ERK inhibitors have been recently reviewed elsewhere [90].

## 3. MEK/ERK-Independent Resistance Mechanisms

In addition to MEK/ERK reactivation, other mechanisms also drive therapy resistance (Figure 2). For example, about 30% of patients develop MEK/ERK-independent resistance to BRAF inhibition in melanoma [55,91]. Numerous MEK/ERK-independent resistance mechanisms to BRAFi have been identified through preclinical studies, although many of these remain to be determined for clinical relevance. Meanwhile, MEK/ERK-independent resistance mechanisms to BRAFi/MEKi combination are much less known. Many of these resistance mechanisms are likely to overlap substantially between BRAFi resistance and BRAFi/MEKi resistance, given the common convergent evolutionary context, i.e., overcoming MEK/ERK inhibition, between them. These mechanisms are summarized below and listed in Table 2 and Table 3.

Loss of phosphatase and tensin homolog (PTEN): The status of PTEN, an important regulator of phosphoinositide 3-kinase (PI3K), is important for determining the propensity of *BRAF*^V600E^ tumor cells to acquire BRAFi resistance through ERK1/2 reactivation. For example, wild-type PTEN-carrying tumors required hyperactivation of ERK1/2 and AKT to resist BRAFi [66], whereas PTEN-inactivated cells required only ERK1/2 activity for resistance [67]. The PTEN status also affects mobilization of the mammalian target of rapamycin (mTOR) pathway for drug resistance. For example, dual BRAF/MEK inhibition initially suppressed the mTOR complex I signaling pathway in melanoma cells in culture and patient-derived tumor xenografts in mice (PDX), but the pathway activity rebounded upon the acquisition of drug resistance in an AKT-dependent manner in *PTEN*-deficient melanoma cells [46]. 

Activation of PI3K/AKT pathway: BRAFi monotherapy or D/T combination therapy frequently led to rebound of AKT phosphorylation at an early stage of treatment in melanomas, suggesting that adaptive resistance involving upregulation of the PI3K/AKT pathway is developed and may affect clinical outcomes of BRAFi therapy [42]. While it is unclear how AKT mediates drug resistance in response to D/T combination therapy, studies of BRAFi resistance demonstrated that it can upregulate embryonic stem cell-expressed Ras (ERAS) to elicit a prosurvival signal though the Bcl-2-associated death promoter (BAD) pathway [92]. The insulin-like growth factor 1 receptor (IGF1R)/PI3K pathway was activated as an acquired resistance mechanism to the BRAFi SB-590885 in *BRAF*^V600E^ melanoma cells [54]. Similarly, IGF1R/Insulin Receptor (IR) expression increased in D/T combination-resistant melanoma cells in correlation with poor patient survival. Moreover, treatment with the IGF1R/IR inhibitor BMS-754807 reduced phosphorylation of AKT but not ERK1/2 [45]. This suggests an involvement of the IGF1R pathway in tumor cell resistance to BRAFi monotherapy and BRAFi/MEKi combination therapy. Of note, these pathways may be monitored to predict patient response to D/T combination. For example, unsupervised clustering of a large cohort of *BRAF*^V600E^ colorectal cancer patients identified molecular subgroups not associated with known clinical characteristics. One subgroup exhibited elevated PI3K/mTOR/AKT/eukaryotic initiation factor 4E-binding protein 1 signaling, whereas the other subgroup dysregulated cell cycle and checkpoint pathways [93]. Interestingly, in response to D/T-combination, the PI3K-upregulated subtype showed higher confirmed response rates, median progression-free survival, and median overall survival, as well as greater immune reactivity than the other group [94].

Activation of survival pathway and altered translation via persistent formation of eukaryotic translation initiation factor 4F (eIF4F) complex: Myeloid leukemia 1 (Mcl-1) overexpression was detected in D/T combination-resistant progressive melanoma biopsies [43]. Indeed, Mcl-1 overexpression conferred resistance to vemurafenib or D/T combination in melanoma cells [43]. Consistent with this, silencing of BH3-only protein conferred resistance to PLX4720 in human melanoma cell lines [63]. As stated below, the apoptotic activator, Bcl-2 modifying factor (BMF), is upregulated upon vemurafenib treatment and may contribute to drug resistance by facilitating eIF4F -mediated translation [58]. Persistent formation of eIF4F complex has been suggested to be a nexus of resistance to anti-BRAF and anti-MEK cancer therapies regardless of whether the resistance mechanisms rely on reactivation of the Raf/MEK/ERK pathway, activation of the PI3K/AKT/mTOR pathway, or modulation of the caspase-dependent apoptotic cascade [58]. This study demonstrated that all these pathways converge on regulating the formation of the eIF4F eukaryotic translation initiation complex, thereby modulating the translation of specific mRNAs. Further, the persistent formation of the eIF4F complex, comprising the eIF4E cap-binding protein, the eIF4G scaffolding protein, and the eIF4A RNA helicase, was associated with resistance to BRAFi, MEKi, and BRAFi/MEKi combination in *BRAF*^V600E^ melanoma, colon, and thyroid cancer cells. The apoptotic activator, BMF, regulated this complex formation by acting on eIF4G cleavage. While vemurafenib induced BMF overexpression, BMF silencing conferred BRAFi resistance and was detected in drug-resistant melanoma cells [58]. Therefore, BMF may be a good surrogate marker indicating the status of eIF4 complex formation and translational activity in tumor cells and, subsequently, drug resistance potential. 

Activation of a G-protein-coupled receptors (GPCR)/cyclic AMP-dependent signaling network: At low frequencies, mutation or overexpression of the transcription factors E26 transformation-specific (ETS) and sterile alpha motif domain containing 4B (SAMD4B) were detected in melanoma relapse after D/T combination therapy [40]. A “gain-of-function” study confirmed the ability of these transcription factors to confer drug resistance in human melanoma cell lines [50]. This study also demonstrated that a cyclic AMP-dependent melanocytic signaling pathway that consists of GPCR, adenyl cyclase, protein kinase A and cyclic AMP response element binding protein (CREB) regulates these and several other transcription factors, including c-FOS, NR4A1, NR4A2, and MITF, which were also segregated to BRAFi-resistance. Indeed, preliminary analysis of *BRAF^V600E^* melanoma biopsies revealed that CREB phosphorylation decreases upon BRAF inhibition but is restored in relapsing tumors [50]. Given that MEK/ERK also regulates these transcription factors, it is conceivable that tumor cells mobilize the cyclic AMP pathway to overcome MEK/ERK deficiency in the context of convergent evolution.

Development of c-JUN-mediated mesenchymal-like phenotype: Vemurafenib resistance in *BRAF*^V600E^ melanoma cell lines is associated with a high abundance of c-JUN and characteristics of a mesenchymal-like phenotype [60]. Early adaptation of tumor cells to the drug was correlated with upregulation of JUN and downregulation of lymphoid enhancer binding factor 1 (LEF1) and sprouty RTK signaling antagonist 4 (SPRY4), and changes in the markers for epithelial-mesenchymal transition (EMT), as determined in cell cultures, xenografts in mice, and patient tumors [60]. Importantly, disrupting the signaling between ERK2 and JUNB and Fos related antigen-1 transcription factors enabled vemurafenib-addicted tumor cells to survive on treatment discontinuation [95], suggesting the involvement of these transcription factors in developing tumor cell addiction to vemurafenib. EMT is an indication of feedback activation of RTK signaling in response to MEK1/2 inhibition in *KRAS*-mutant lung cancers [96], and it has been proposed as a marker for MEKi resistance [97]. 

Activation of signal transducer and activator of transcription 3 (STAT3) signaling pathway: The EGFR-SRC family kinase (SFK)-STAT3 pathway is involved in vemurafenib resistance of melanoma. For example, increased EGFR and SFK activity was detected in association with increased tumor cell proliferation, invasion, and metastasis in tumor biopsies from patients with intrinsic or acquired vemurafenib resistance, and EGFR inhibitors cooperated with BRAFi to block the growth of the resistant cells in vitro and in vivo [64]. In line with this, interleukin 6 (IL6) secreted by cancer-associated fibroblasts can induce EMT and drug resistance of esophageal adenocarcinoma [98]. Given that IL6 activates STAT3 via its canonical effector janus kinase (JAK), activation of STAT3 may also underlie the EMT-mediated drug resistance [99]. 

Upregulation of Hippo and yes-associated protein 1 (YAP1) signaling: YAP was identified as a vemurafenib resistance gene by shRNA-mediated loss of function screening in the *BRAF*^V600E^ NSCLC line HCC364 [61]. In this study, combined YAP inhibition with RAF or MEK inhibition induced synthetic lethality not only in BRAF tumor cells but also in RAS tumor cells [61]. This study also proposed YAP1 upregulation as a biomarker of poor initial response to BRAF and MEK inhibition in *BRAF*^V600E^ tumor patients [61]. The significance of YAP1 is supported by other studies that also identified YAP1 as a biomarker and a drug resistance mediator [72,100,101,102]. 

Reprogramed metabolic processes: Oncogenic BRAF can regulate oxidative metabolism via peroxisome proliferator-activated receptor γ coactivator 1-α (PGC1α), whose transcription is directly regulated by microphthalmia-associated transcription factor (MITF), a target of BRAF for negative regulation [103]. *BRAF*^V600E^ melanoma cells enhance aerobic glycolysis while suppressing mitochondrial respiration by downregulating these transcription factors [104]. Consistently, inhibition of BRAF or MEK1/2 increases oxidative phosphorylation and mitochondrial biogenesis in *BRAF*-mutated melanoma cells through MITF and PGC1α upregulation. Moreover, melanoma cells intrinsically resistant to or adapted to BRAF inhibition exhibit lower basal levels of mitochondrial biogenesis and addiction to oxidative phosphorylation, respectively [103,105]. Similar to melanoma cells, trametinib-resistant *KRAS*-mutant NSCLC cells exhibit increased mitochondrial respiration [106]. Notably, these metabolic alterations have been proposed as a lineage program in melanoma cells resistant to BRAF/MEK inhibition [50,92,95,103,107,108,109,110]. Indeed, MITF alteration is a part of the genetic landscape of clinical resistance to BRAF inhibition in metastatic melanoma [109]. This distinct phenotype plasticity of tumor cells in response to BRAF/MEK inhibition is partly regulated epigenetically—an in-depth review of the epigenetic mechanisms underlying the drug resistance is available elsewhere [111]. Another example of cell lineage-specific drug resistance is found in thyroid cancer. *RAS* or *RAF* mutations leading to malignant thyroid epithelium transformation are accompanied by dedifferentiation and a decrease in the sodium-iodide symporter (SLC5A5) expression, which results in resistance to radioactive iodine therapy. Indeed, D/T combination, but not dabrafenib alone, upregulated sodium-iodide symporter expression in patient-derived thyroid tumor cells in culture, suggesting the possibility that D/T combination may increase tumor cell uptake of radioactive ^131^I [112]. Intriguingly, this effect was more significant in tumor cells from younger patients, implicating the involvement of a developmental biological aspect. This concept has been recently proven in a clinical trial [113]. An in-depth review of the use of MAPK pathway inhibitors in thyroid cancer is available elsewhere [114].

## 4. Co-Evolution of Intra-Tumoral Immunity 

**Figure 3:**
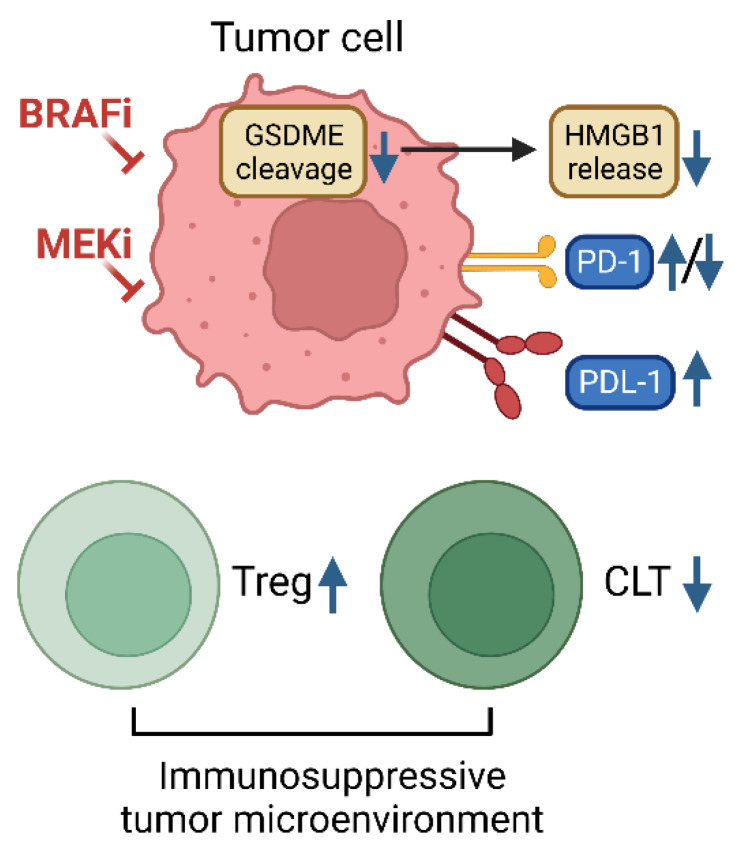
Immune-modulating effects of BRAFi and MEKi. Tumor cells can develop resistance to BRAFi and MEKi by creating an immunosuppressive tumor microenvironment. Drug-resistant tumor cells fail to undergo pyroptosis induced by BRAFi and MEKi, exhibiting decreased GSDME cleavage and high mobility group box 1 (HMGB1) release. They also display dysregulated PD-1/PDL-1 expressions which affects antitumor immune responses, including increased regulatory T cells (Treg), and decreased cytotoxic T cells (CLT). This figure was created with biorender.com.

Increasing evidence suggests that BRAF and MEK inhibitors have immune-modulating effects and can enhance antitumor immunity (illustrated in Figure 3). For example, advanced melanoma patients treated with BRAFi or BRAFi/MEKi combination exhibited increased expression of programmed cell death 1 (PD-1) and its ligand, PD-L1 [115]. Dual BRAF/MEK inhibition also expanded memory and activated/exhausted CD8+ T cells, which was required for durable tumor regression to be elicited [116]. These suggest that BRAF/MEK inhibitors and an immune-therapeutic modality can synergize for tumor suppression, leading to clinical trials (Table 4). Indeed, a combination of D/T with the PD-1 antibody pembrolizumab prolonged antitumor responses and progression-free survival of *BRAF*-mutant melanoma patients [117,118]. A combination of D/T and spartalizumab, another PD-1 antibody, was also tested for *BRAF* melanoma patients in a phase III trial, but overall survival benefit was not observed [119,120]. However, in contrast, the spartalizumab combination with D/T showed survival benefit potential for *BRAF*-mutant colorectal cancer patients, and a single-cell RNA sequencing analysis of this cohort revealed that more effective induction of tumor cell-intrinsic immune programs and MEK/ERK inhibition is associated with better clinical outcomes [121]. As such, post-hoc analyses of different trials are required to identify tumor type-specific biomarkers for the precise selection of patients for the triple drug combination, as recently proposed [122]. For example, dual BRAF/MEK inhibition induces cleavage of pyroptosis marker gasdermin E (GSDME) and intra-tumoral T cell infiltration, but BRAFi/MEKi resistance attenuates these responses in melanoma [48]. Consistently, tumor biopsies showed CD8+ T cell deficiency and exhaustion and PD-1 downregulation in BRAFi- or BRAFi/MEKi-resistant melanoma [72], and vemurafenib or vemurafenib/trametinib combination impaired T cell activation [51]. A better understanding of tumor-specific differences in a molecular mechanism might help advance the strategy to combine immune checkpoint inhibitors with BRAF/MEK inhibitors. A more extensive review in this area is available elsewhere [123].

## 5. Future Perspectives and Conclusions

Precision medicine cancer treatment has greatly advanced by accumulating data on the genotype–phenotype relationship of various oncogenic mutations. Targeting BRAF and MEK1/2 in combination is now a key therapeutic strategy for *BRAF* tumors, as D/T combination therapy showed efficacy in a broad spectrum of tumors. Nonetheless, significant challenges remain for D/T combination therapy. First, certain *BRAF* tumors have an intrinsic ability to rapidly adapt to the presence of these drugs by bypassing drug effects via rewired signaling, metabolic, and regulatory networks. Second, almost all tumors initially responsive to D/T combination eventually acquire therapy resistance via an additional genetic/epigenetic alteration(s). Overcoming these challenges requires identifying the molecular background of a tumor type, other than *BRAF* mutations, that also determines clinical outcomes. Indeed, many potential gene signatures of MEK/ERK functional outputs have been identified from therapy-resistant tumor cells. For example, a 13-RAS effector gene signature has been identified to predict the existence of compensatory signaling in selumetinib-resistant tumor cells [124]. Several of the genes in this signature have been functionally validated [70]. A 147-gene expression signature was also identified to predict *RAS*-mutant tumor responsiveness to PI3K and RAS pathway inhibition [125]. Multiple somatic mutations in patients have also been detected in association with therapy resistance [39,40]. In vitro ‘gain- or loss-of-function’ studies have been conducted to identify many candidate resistance genes [50,61]. The status of these genes might need to be analyzed comparatively in patient exome and RNA sequencing data from clinical trials. Data analysis should also consider the off-target effect of a drug. For example, dabrafenib but not vemurafenib can inhibit NIMA (Never In Mitosis Gene A)-related kinase and cyclin-dependent kinase-16 in addition to BRAF [126]. 

Identifying reliable prognosis markers through active correlative and functional analysis of molecular alterations associated with clinical outcomes will enable the establishment of a reliable guideline for companion diagnostics. Whether similar across tumor types or tumor type-specific, knowledge of these alterations is expected to refine patient selection and improve clinical outcomes, eventually providing the maximal benefit of BRAF/MEK/ERK targeted therapies.

## Figures and Tables

**Table 1:** The rates of *BRAF^V600^* mutations in different tumor types.

Tumor Types	Rates (%)					Reference
	* E	K	D	R	M	
Melanoma	~50	~9	~0.04	~0.4	~0.1	[8,9,10,11]
Thyroid carcinoma	~40	N.D.	N.D.	N.D.	N.D.	[12,13]
Colorectal cancer	~10	N.D.	N.D.	N.D.	N.D.	[14]
Lung adenocarcinoma	~5	N.D.	N.D.	N.D.	N.D.	[15]
Cholangiocarcinoma	~13	N.D.	~3	N.D.	N.D.	[16]
Pleomorphic xanthoastrocytomas	~65	N.D.	N.D.	N.D.	N.D.	[17]
Gangliogliomas	~50	N.D.	N.D.	N.D.	N.D.	[17,18]
Desmoplastic Infantile Ganglioglioma/Astrocytoma	~25	N.D.	~19	N.D.	N.D.	[19]
Pilocytic astrocytomas	~9	N.D.	N.D.	N.D.	N.D.	[16]
Oligodendrogliomas	~2	N.D.	N.D.	N.D.	N.D.	[18]
Hairy cell leukemia	~100	N.D.	N.D.	N.D.	N.D.	[20]
Multiple myeloma	~9.3	N.D.	N.D.	N.D.	N.D.	[21,22]

* Indicated is the amino acid that switches V600; N.D. not yet detected.

**Table 2:** Resistance mechanisms to combination therapy of BRAF and MEK1/2 inhibitors.

Drugs	Tumor Types	Source of Study	Alterations for Resistance	Resistance Types	Consequence	Reference
* Dabra/Tram	Melanoma	Patient biopsy	*BRAF* amplification, *NRAS* mutations, *MEK2*^C125S^	Acquired	ERK1/2 reactivation	[39]
Dabra/Tram	Melanoma	Patient biopsy	*BRAF* splicing isoform lacking exons 2-10, *MEK2*^Q60P^, Somatic mutations of *ETS*, *SAMD4B*	Acquired	ERK1/2 reactivation	[40]
Dabra/Tram	Melanoma	Patient biopsy	Activating *BRAF* in-frame deletion	Acquired	ERK1/2 reactivation	[41]
Dabra/Tram	Melanoma	Patient biopsy, cell lines	*AKT1*^Q79K^ that activates PI3K-AKT signaling, PDGFR- β upregulation	Adaptive	MEK/ERK-independent resistance	[42]
Dabra/Tram	Melanoma	Patient biopsy	*MCL-1* overexpression, activation of survival pathway	Adaptive	MEK/ERK-independent resistance	[43]
Dabra/Tram	Colorectal cancer	Patient biopsy	*KRAS* amplification, *BRAF* amplification, *MEK1*^F53L^	Acquired	ERK1/2 reactivation	[38]
Dabra/Tram	Colorectal cancer	Patient biopsy	*KRAS*^G12C^, *BRAF*^V600E^ allele frequency increase	Acquired	ERK1/2 reactivation	[44]
Dabra/Tram	Melanoma	Cell lines, PDX model, biopsy	Increase of IGF1R/IR expression	Acquired	MEK/ERK-independent resistance	[45]
PLX4720/PD0325901	Melanoma	Cell lines, PDX model	Rebound of mTOC1 pathway	Acquired	AKT or ERK contributes to the activation of mTORC1 depending on PTEN status	[46]
PLX4720/Tram Dabra/Tram	Melanoma	Cell lines, PDX model	Upregulation of ATF4	Acquired	ERK1/2 reactivation	[47]
PLX4720/PD0325901	Melanoma	Synergetic mouse model, cell lines,	Failed to induce GSDME, decreased intra-tumoral T cell infiltration	Acquired	MEK/ERK-independent resistance	[48]
BRAFi/EGFRi (dabrafenib + panitumumab), BRAFi/EGFRi/MEKi (dabrafenib + panitumumab + trametinib)	Colorectal cancer	Patient biopsy, cell lines	One or more RAS mutations (*KRAS* or *NRAS*)	Acquired	ERK1/2 reactivation	[49]
PLX4720+ AZD6244	Melanoma	Gain of function screen, Patient biopsy	GPCR-PKA-cAMP, CREB phosphorylation	Adaptive	MEK/ERK-independent resistance	[50]
PLX4720+ AZD6244	Melanoma	Gain of function screen, patient biopsy	c-Fos, NR4A1, NR4A2, MITF, activation of MEK/ERK downstream effectors	Intrinsic, adaptive, acquired	MEK/ERK-independent resistance	[50]
Vemurafenib only or Vemurafenib/Tram	Melanoma	Cell lines, Patient biopsy	Decreased ability to induce IFNγ release by CD8+ TILs	Acquired	Decreases T cell activation	[51]
Vemurafenib only or Vemurafenib/Tram	Melanoma	Cell lines, Patient biopsy	Decreased TOP1 expression	Acquired	Unclear	[52]

* Dabrafenib/trametinib combination.

**Table 3:** Resistance mechanisms to BRAF or MEK1/2 inhibitors.

Drug	Tumor Types	Source of Study	Alterations for Resistance	Resistance Types	Consequence	Reference
Vemurafenib	Melanoma	Patient biopsy, cell lines	PDGFR-β upregulation, *NRAS*^Q61K^	Acquired	ERK1/2 reactivation	[53]
Dabrafenib	Melanoma	Cell lines	*MEK1*^K59del^, *NRAS*^Q61K^ and/or *NRAS*^A146T^ with and without *MEK1*^P387S^	Acquired	ERK1/2 reactivation	[37]
SB590885	Melanoma	Patient biopsy, cell lines	IGF1R-PI3K-AKT activation	Acquired	MEK/ERK-independent resistance	[54]
Dabrafenib or vemurafenib	Melanoma	Patient biopsy	*RAS* mutations, mutant *BRAF* amplification, and alternative splicing	Acquired	ERK1/2 reactivation	[55]
Dabrafenib or vemurafenib	Melanoma	Patient biopsy	*AKT1*^E17K^ and *AKT1*^Q79K^	Acquired	MEK/ERK-independent resistance	[55]
Vemurafenib	Melanoma	Cell lines	FGFR3-Ras activation	Acquired	ERK1/2 reactivation	[56]
Vemurafenib	Melanoma	Cell lines	SHOC-2/Sur-8 expression for N-Ras/C-Raf interaction	Acquired	ERK1/2 reactivation	[57]
Vemurafenib	Melanoma	Cell lines	Bcl-2 modifying factor (BMF) downregulation, increased eIF4F complex formation, reprogrammed translation	Acquired, adaptive	MEK/ERK-independent resistance	[58]
Vemurafenib	Melanoma	Cell lines	Relief of feedback inhibition of mitogenic signaling	Adaptive	ERK1/2 reactivation	[59]
Vemurafenib	Melanoma	Patient biopsy, Cell lines	c-JUN upregulation, LEF1 and SPRY4 downregulation, activation of downstream effector	Acquired, adaptive	MEK/ERK-independent resistance	[60]
Vemurafenib	NSCLC, Melanoma	Cell lines, Patient biopsy	YAP upregulation, activation of downstream effectors	Intrinsic, adaptive	MEK/ERK-independent resistance	[61]
PLX4720	Melanoma	Gain of function screen	MAP3K8/COT/TPL-2	Secondary tumor development	ERK1/2 reactivation	[62]
PLX4720	Melanoma	Cell lines	BH-3 only protein silencing, activation of survival pathway	Acquired	MEK/ERK-independent resistance	[63]
Vemurafenib	Melanoma	Cell lines, Patient biopsy	EGFR-SFK-STAT3, activation of downstream effector	Acquired, adaptive	ERK1/2 reactivation	[64]
Vemurafenib	Melanoma	Cell lines	Activation of MAPKs and the PI3K pathways, enhanced NRAS expression	Acquired	Activation of all the three MAPKs, ERK, JNK, and p38	[65]
Vemurafenib	Melanoma	Cell lines	Upregulated AXL in PTEN wild-type cells	Acquired	Hyperactivation of AXL/AKT and ERK pathways	[66]
Vemurafenib	Melanoma	Cell lines	Upregulated PERK in PTEN-inactivated	Acquired	Hyperactivation of ERK pathway	[67]
Vemurafenib	Thyroid cancer	Cell lines	ERBB/HER3 transcription, autocrine secretion of neuregulin 1	Adaptive	ERK1/2 reactivation	[68]
Vemurafenib	Colorectal cancer	Cell lines	EGFR activation	Adaptive	ERK1/2 reactivation	[69]
Selumetinib	Colorectal cancer	Cell lines	KRAS or BRAF amplification	Acquired	ERK1/2 reactivation	[70]
Selumetinib	Melanoma	Patient biopsy	*MEK1* ^P124L^	Acquired	ERK1/2 reactivation	[71]
Selumetinib	Melanoma	Patient biopsy, cell lines	c-MET up-expression, LEF1 down-expression, YAP1 signature enrichment	Acquired	ERK1/2 reactivation	[72]
Selumetinib	Colorectal cancer	Cell lines	BRAF amplification	Acquired	ERK1/2 reactivation	[73]

**Table 4:** Clinical trials testing the combination of BRAF/MEK inhibition and immunotherapy.

Drugs	Tumors	Outcomes	Reference
Dabra/Tram and Pembrolizumab	Melanoma	Improved patient survival and antitumor responses	[117,118]
Dabra/Tram and Spartalizumab	Melanoma	No significant overall survival differences	[119,120]
Dabra/Tram and Spartalizumab	Colorectal cancer	Improved patient survival and antitumor responses	[121]

## Data Availability

Not applicable.
